# Supplementary material for: NMR Studies of Two Lysine Based Dendrimers with Insertion of Similar Histidine-Arginine and Arginine-Histidine Spacers Having Different Properties for Application in Drug Delivery
Source: Int J Mol Sci. 2023 Jan 4;24(2):949. doi: 10.3390/ijms24020949 (PMC9866564; doi:10.3390/ijms24020949)
Supplement: Supplementary file 1 [file ijms-24-00949-s001.zip › ijms-2097272-supplementary.pdf]

SUPPLEMENTARY MATERIALS

NMR studies of peptide dendrimers with Histidine-Arginine (Lys-His-Arg) and Arginine-Histidine (Lys-Arg-His) Spacers

Nadezhda N. Sheveleva,[1] Irina I. Tarasenko,[2] Mikhail A. Vovk,[1] Mariya E. Mikhailova,[1] Igor M. Neelov,[3] Denis A. Markelov[1]

[1]*Saint Petersburg State University, 7/9 Universitetskaya nab, 199034 Saint Petersburg, Russia*
[2]*Institute of Macromolecular Compounds, Russian Academy of Sciences, Bolshoi Prospect 31, V.O., 199004 Saint Petersburg, Russia*
[3]*Saint Petersburg National Research University of Information Technologies, Mechanics and Optics (ITMO University), Kronverkskiy pr. 49, 197101 Saint Petersburg, Russia*

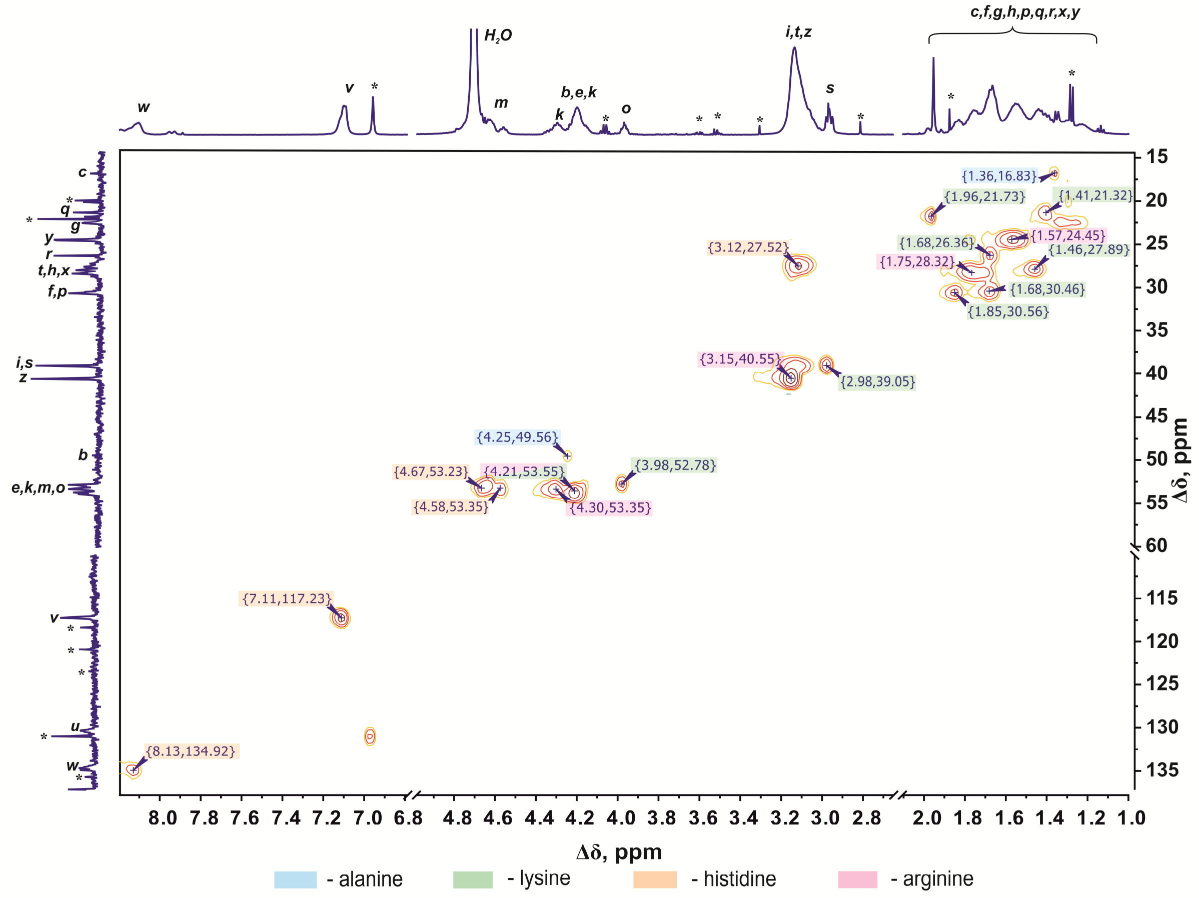

Figure S1. *^{1}H-^{13}C HSQC NMR spectrum of the Lys-Arg-His dendrimer at 298 K. The peaks from small molecular weight impurities are marked by*.*

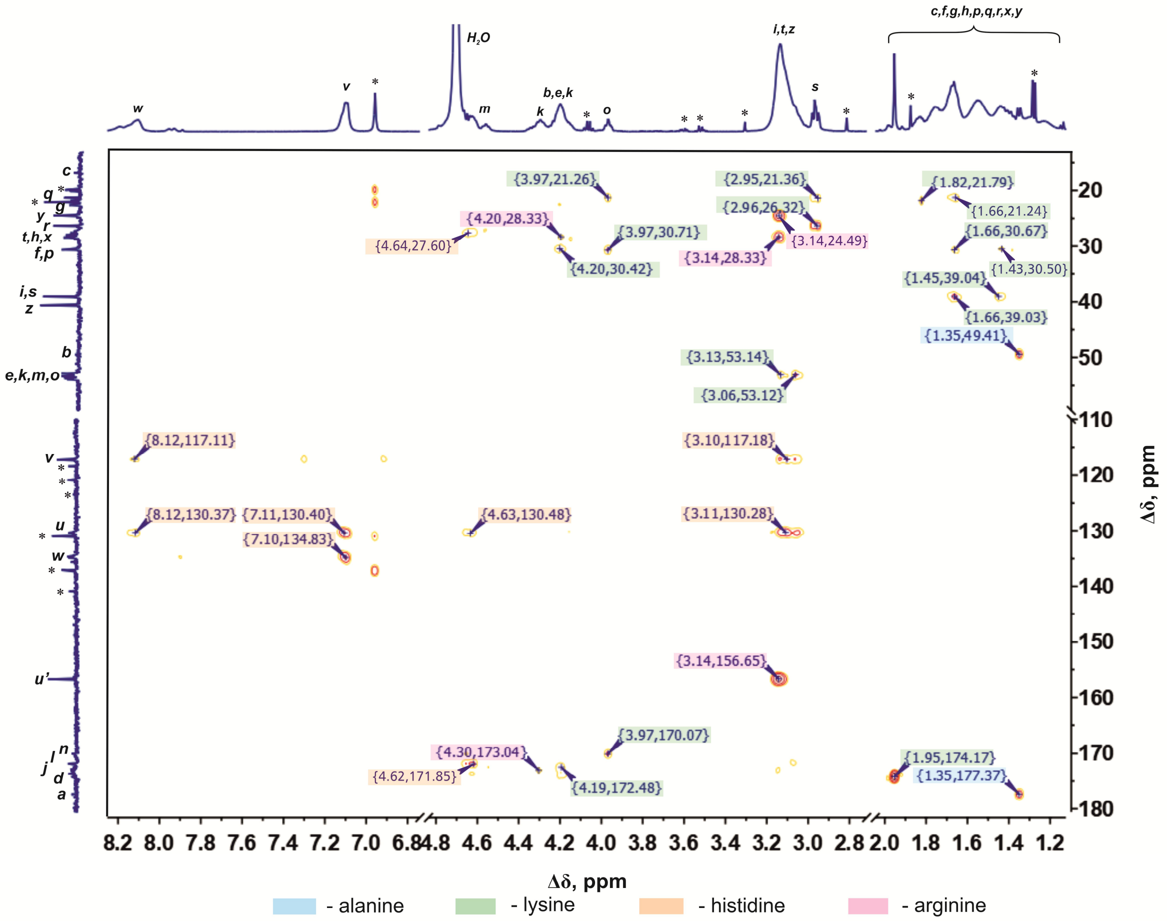

Figure S2. *^{1}H-^{13}C HMBC NMR spectrum of the Lys-Arg-His dendrimer at 298 K. The peaks from small molecular weight impurities are marked by*.*

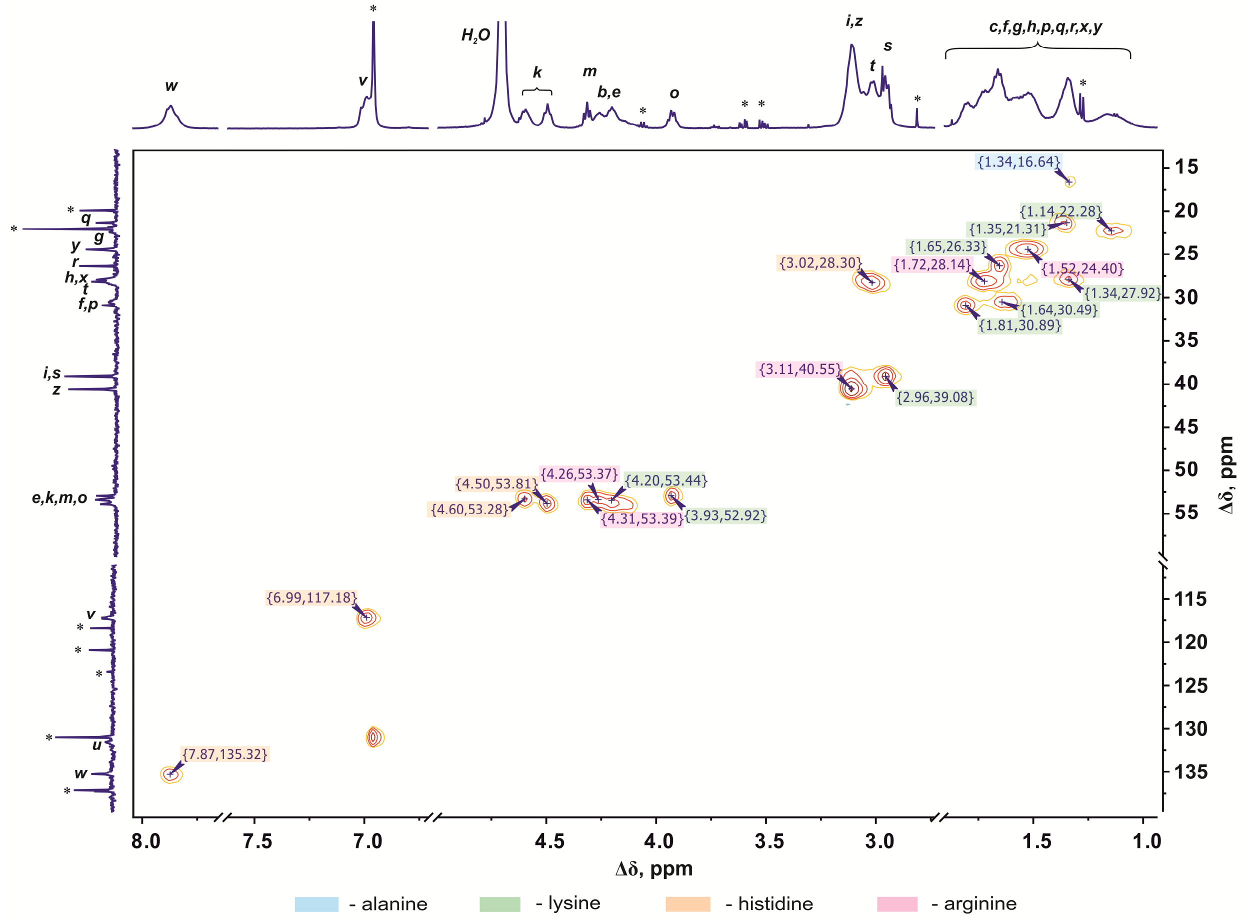

Figure S3. *^{1}H-^{13}C HSQC NMR spectrum of the Lys-His-Arg dendrimer at 298 K. The peaks from small molecular weight impurities are marked by*.*

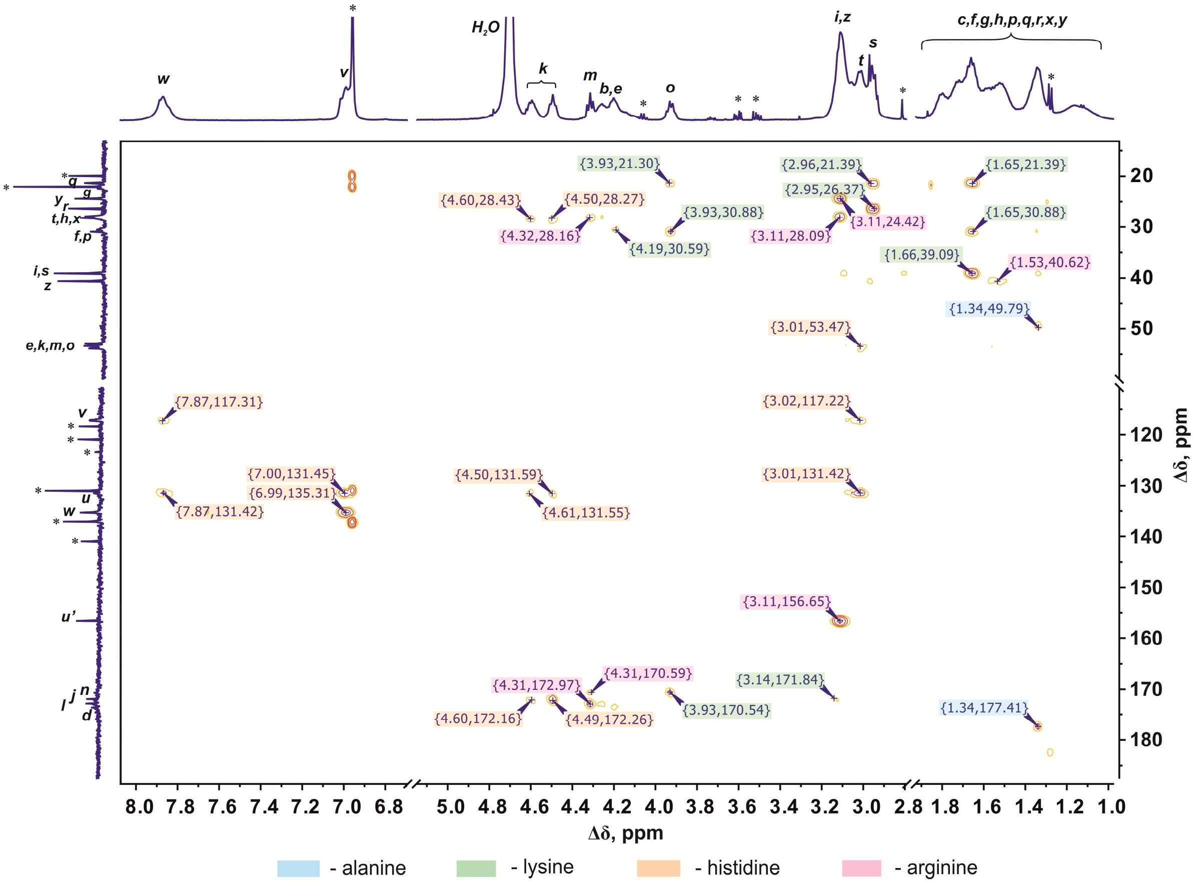

Figure S4. *^{1}H-^{13}C HMBC NMR spectrum of the Lys-His-Arg dendrimer at 298 K. The peaks from small molecular weight impurities are marked by*.*
